# Desmoplastic Fibroma of the Ulna Bone: A Case Report of a 16-Year-Old Girl

**DOI:** 10.7759/cureus.79050

**Published:** 2025-02-15

**Authors:** Abdullah S Alaboudi, Mustafa Alrawi, Osama AlShaya, Khaled AlAbbasi

**Affiliations:** 1 Orthopaedic Surgery, King Fahad Medical City, Riyadh, SAU

**Keywords:** bone tumor, desmoplastic fibroma, distal ulna, histopathology, surgical management

## Abstract

Desmoplastic fibroma (DF) is a rare, benign, yet locally aggressive bone tumor, typically affecting long bones in young individuals. Its occurrence in the ulna is extremely rare. This case report highlights the diagnostic challenges of a 16-year-old female presenting with DF of the ulna. The report emphasizes individualized treatment and long-term follow-up and raises awareness of DF in atypical locations. This patient presented with a two-month history of mild, activity-related pain in the distal forearm. Imaging revealed an expansile lytic lesion in the distal ulna, characterized by cortical thinning and breaches, consistent with desmoplastic fibroma (DF). Routine blood tests were normal, and histopathology confirmed spindle fibroblasts in a collagenous stroma with dilated vascular channels, confirming DF. The patient underwent intralesional curettage, bioceramic cement application, and plate and screw fixation. Postoperatively, she experienced mild surgical site pain but retained full wrist motion and grip strength. At six months, the patient showed no recurrence and good functional outcomes. This case highlights the rarity of desmoplastic fibroma in the ulna, emphasizing the challenges of diagnosis and management. Accurate diagnosis through imaging and histopathology, combined with individualized surgical treatment, preserved function and prevented reoccurrence. It underscores the need for long-term follow-up to ensure successful outcomes and adds valuable insight to the limited literature on this condition.

## Introduction

Desmoplastic fibroma (DF) is a rare, locally aggressive, non-metastasizing, benign primary bone tumor, making up less than 0.1% of all bone tumors [1]. It is marked by fibroblastic proliferation that infiltrates nearby bone tissue, causing bone destruction and sometimes leading to serious functional problems. DF usually affects long bones, with the most common site being the mandible, followed by the femur and pelvis, and is most often found in children and young adults [2]. However, its occurrence in the ulna bone is extremely rare, with only a few cases reported in the literature [2,3].

The clinical relevance of DF lies in its diagnostic and treatment challenges. Its nonspecific symptoms, like localized pain or swelling, can mimic other bone pathologies, which often delays diagnosis [4]. Furthermore, DF is associated with significantly high recurrence rates despite aggressive management. Radiographic findings are also not always definitive, as DF often appears as a lytic lesion with cortical thinning [5], which can raise concern for malignancy. The accurate diagnosis of DF relies heavily on histopathology, and failure to diagnose early can lead to local progression and functional limitations [6].

This case report presents a rare instance of DF in the ulna bone of a 16-year-old girl, underscoring its rareness and the diagnostic challenge it poses. With limited literature on desmoplastic fibroma occurring in atypical sites, this report offers insights into the diagnostic procedures and surgical management of the condition. The findings highlight the importance for clinicians to consider the possibility of DF in unusual locations. This report enhances understanding of DF, emphasizes long-term follow-up, and advocates individualized treatment to improve outcomes and recurrence management.

## Case presentation

A 16-year-old girl was referred to our tertiary care center after being diagnosed with a destructive right ulna bone lesion detected on plain radiographs. The patient's main complaint was pain, localized to the distal forearm, occurring mostly with heavy activities. There was no history of swelling, redness, fever, or any systemic symptoms. The pain was mild but had been getting progressively worse over the past two months prior to presentation. She had no significant medical history, no previous trauma, and no known chronic illness. She also had no family history of bone or CT disorders. Her general health and development were otherwise uneventful.

On physical examination, there was no apparent swelling, deformity, or any skin changes over the right forearm. Mild tenderness was felt on palpation of the distal ulna, but no palpable mass was found. The wrist had a full range of motion, including pronation and supination, with symmetrical grip strength on both sides. The neurovascular examination was normal, with intact circulation and nerve function. Routine blood tests, including complete blood counts, inflammatory markers, and biochemistry, were normal.

**Figure 1:**
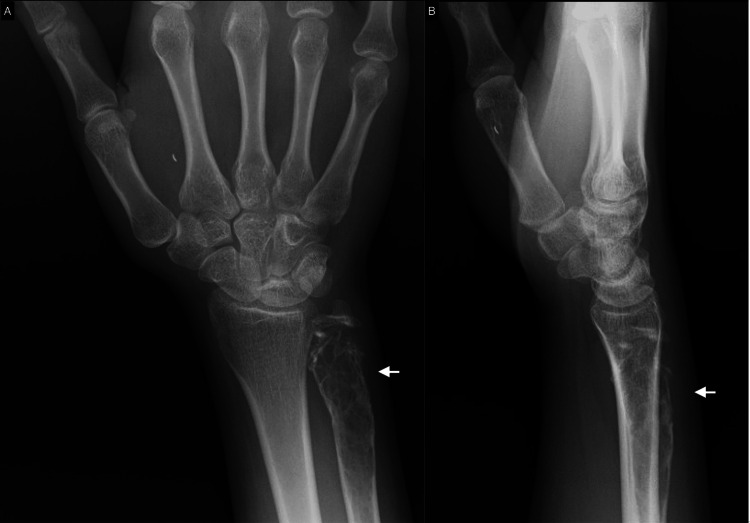
Anteroposterior (A) and lateral (B) views of the distal ulna showing a 6.4-cm long lesion in the distal ulna

**Figure 2:**
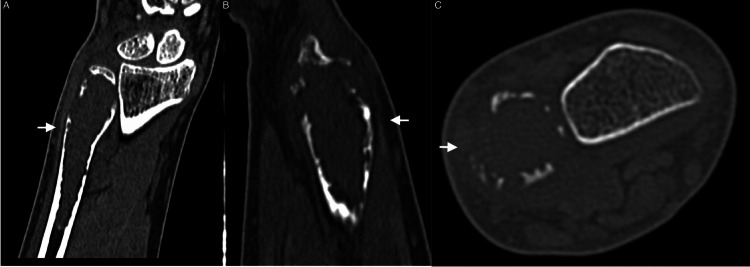
Coronal (A), sagittal (B), and axial (C) CT scan of the left distal ulna showing a lytic lesion in the ulnar metaphysis, extending into the epiphysis and diaphysis and measuring 1.5 x 1.6 x 6.4 cm.

**Figure 3:**
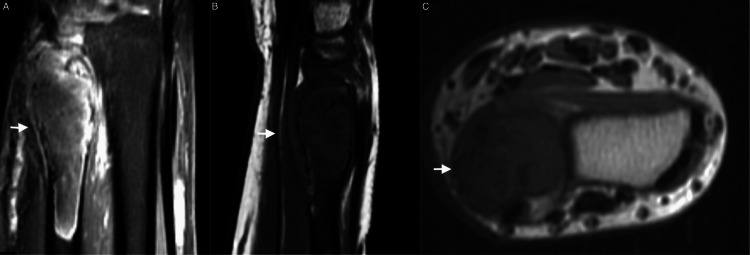
T2 coronal (A) and T1 sagittal (B) and axial (C) MRI showing an expansile lesion centered at the distal ulna with cortical thinning and minimal bone marrow edema

Plain radiographs revealed a distal ulna lytic lesion with cortical thinning, breaches, and intact joint alignment, consistent with a benign but locally aggressive lesion like desmoplastic fibroma (Figure 1). CT imaging of the distal ulna revealed an expansive lytic lesion located in the metaphysis, extending into the epiphyseal and diaphyseal regions. The lesion is marked by significant cortical thinning and multiple areas of cortical breakthrough without displacement. No aggressive periosteal reactions or additional lesions were seen. The lesion measured 1.5 x 1.6 x 6.4 cm in its transverse, anteroposterior, and cranio-caudal dimensions (Figure 2). The MRI revealed an expansile lesion centered at the distal ulna epiphysis extending into the diaphysis. The lesion demonstrated significant cortical thinning and a heterogeneous low signal on T1 and T2 sequences with post-contrast enhancement. (Figure 3).

After a detailed discussion with the family, the patient underwent intralesional curettage, bioceramic cement application, and plate and screw fixation. Postoperatively, the patient reported mild surgical site pain but had intact wrist movement and good grip strength. Pain management included a wrist splint and occupational therapy for the range of motion and strengthening exercises.

Histopathological examination confirmed a spindle cell lesion composed of mature fibroblasts embedded in abundant collagen with thin-walled, dilated vascular channels, consistent with desmoplastic fibroma. Surgical margins were reported to be clear.

The final diagnosis was a desmoplastic fibroma of the distal ulna. At six months, the patient had mild pain, full motion, no recurrence, and continued follow-up. Postoperative X-rays show stable fixation with a plate and screws, with no complications, hardware issues, or pathological fractures.

## Discussion

Desmoplastic fibroma (DF) is a rare, benign, but locally aggressive bone tumor, typically affecting individuals in their early years and commonly involving the long bones, pelvis, or mandible. Its occurrence in the ulna is exceptionally rare. This discussion highlights the clinical, radiological, and histopathological features of DF in this case and compares them with existing literature. Our patient, a 16-year-old girl, presented with a two-month history of mild, activity-related forearm pain localized to the distal ulna. The absence of swelling, systemic symptoms, or deformity is consistent with typical presentations of DF. In a study by Nedopil et al., the most common symptom of DF was reported as localized pain, often associated with functional limitations [4]. Swelling or palpable masses, although reported in some cases [7], are less frequent, particularly in the early stages. Eyesan et al. show that desmoplastic fibroma is initially asymptomatic but can present with pain, swelling, joint effusion, or rare pathological fractures [2]. Our case aligns with the literature, as the patient exhibited only mild tenderness without deformity or functional compromise. However, Razavipour et al. show that desmoplastic fibroma can be tender and have painful motion but normal passive range and an unremarkable neurologic exam [8].

Moreover, the rarity of DF in the ulna complicates diagnosis. According to Jaffe and Guerrero et al., the tumor is often misdiagnosed due to its non-specific symptoms and radiological overlap with other lesions, such as aneurysmal bone cysts, fibrous dysplasia, or low-grade sarcomas [9,10]. In our case, the initial differential diagnosis based on imaging included an aneurysmal bone cyst, reflecting this diagnostic challenge.

Radiological findings are key to identifying DF, although they are not definitive without histopathological correlation [11]. Preoperative X-rays of our patient revealed a lytic lesion in the distal ulna with cortical thinning and breaches, consistent with features described in previous studies. According to Tanwar et al. and Karimi et al., the classic radiological appearance of DF includes an expansile, lytic lesion with well-defined margins, cortical thinning, and occasional cortical breakthrough [1,6]. Our CT findings of a lesion measuring 1.5 x 1.6 x 6.4 cm with significant cortical thinning and multiple cortical breaches are consistent with this pattern.

Notably, MRI gave further characterization, showing heterogeneous low T1 and T2 signals with post-contrast enhancement and minimal bone marrow edema. These features match findings from Kim et al., who noted that MRI often shows lesions with low signal intensity on T1-weighted images and variable signals on T2-weighted images, depending on fibrous contents and collagen density [12]. The lack of extraosseous soft tissue involvement in our case is reassuring and typical of benign lesions, distinguishing DF from malignancies such as low-grade osteosarcoma or fibrosarcoma.

Moreover, histopathology remains the gold standard for confirming the diagnosis of DF. In our case, the biopsy revealed spindle-shaped fibroblasts embedded in a collagenous stroma with thin-walled, dilated vascular channels, findings consistent with DF. These features are consistent with Kahraman et al., which showed unencapsulated, infiltrative tumors with spindle fibroblastic cells, collagenized matrix, no hard tissue, atypia, mitosis, or necrosis [13]. The absence of cellular atypia, mitotic activity, and necrosis further supported the benign nature of the lesion. In comparison, Böhm et al. analyzed 32 cases of DF and noted similar histopathological features, emphasizing the importance of clear surgical margins to prevent recurrence [14]. Our case confirmed clear surgical margins, reducing the likelihood of recurrence, which is a critical factor in the management of DF. 

Notably, DF treatment involves surgical excision; our patient underwent intralesional curettage, bioceramic cement application, and fixation to preserve ulna integrity and wrist function, given well-defined margins and no significant soft tissue extension. In a study by Myilvahanan et al., intralesional curettage with bone grafting was shown to be effective for small, localized lesions with low recurrence risk [15]. A retrospective study of 36 skeletally immature patients with locally aggressive bone tumors found no significant differences in reoperation rates, local recurrence, growth-related complications, adjacent joint arthrosis, or postoperative pain between groups treated with polymethylmethacrylate (PMMA) cement and bone grafts; however, postoperative fractures were observed only in the bone graft group [16]. Additionally, a systematic review and meta-analysis reported significantly lower recurrence rates with bone cement (11.2%) compared to bone grafting (27.56%) in cases of giant cell tumors [17]. However, the literature highlights a recurrence rate of 10% to 38% for intralesional curettage compared to lower rates with wide resection [18]. Our six-month follow-up revealed no recurrence, demonstrating the success of our approach, though a longer follow-up is essential.

Postoperatively, our patient retained full wrist motion and good grip strength, with only mild residual pain at six months. Functional outcomes are an important aspect of DF management, as aggressive resection can lead to significant morbidity. According to Bohm et al., preserving function is particularly critical in young patients, where growth potential and physical activity are key considerations [14]. Our case underscores the importance of a balanced approach that prioritizes both oncological control and functional preservation.

## Conclusions

Desmoplastic fibroma (DF) is a rare, locally aggressive, but non-metastasizing bone tumor arising from fibroblastic proliferation, often presenting with bone destruction and a high recurrence rate. It accounts for less than 0.1% of all bone tumors, primarily affecting young adults, and poses diagnostic challenges due to its radiographic resemblance to malignant bone lesions. This case of a 16-year-old girl with DF of the distal ulna highlights the importance of clinical, radiological, and histopathological evaluations for an accurate diagnosis. Treatment with intralesional curettage, bioceramic cement, and fixation with plate and screws is associated with acceptable successful functional rates but with high recurrence rates.
